# Basophils absence predicts poor prognosis and indicates immunosuppression of patients in intensive care units

**DOI:** 10.1038/s41598-023-45865-y

**Published:** 2023-10-28

**Authors:** Xiao Chen, Xiaofeng Zhu, Huichang Zhuo, Jiandong Lin, Xian Lin

**Affiliations:** 1 Department of Intensive Care Unit and The Clinical Key Specialty of Fujian Province, First Affiliated Hospital of Fujian Medical University, Fuzhou, Fujian China; 2 Department of Intensive Care Unit, National Regional Medical Center, Binhai Campus of the First Affiliated Hospital, Fujian Medical University, Fuzhou, Fujian China; 3 Department of Oral Maxillo-Facial Surgery, The First Affiliated Hospital of Fujian Medical University, Fuzhou, Fujian China; 4 Shenzhen Key Laboratory of Immunity and Inflammatory Diseases, Peking University Shenzhen Hospital, Shenzhen Peking University-The Hong Kong University of Science and Technology Medical Center, Shenzhen, Guangdong China

**Keywords:** Biomarkers, Outcomes research, Translational research

## Abstract

Immune cells and immunity are associated with the prognosis of patients with critical illness. Here, medical records retrospectively extracted from the Medical Information Mart for Intensive Care IV were used for screening an immune-related biomarker in intensive care units (ICU) patients and applied for validating the identified indicator in septic patients. In this work, the count of innate immune cells, basophils, harbored a superior role in predicting ICU patients’ prognosis compared with those of other blood immune cells (OR 0.013, 95% CI 0.001, 0.118, *P* < 0.001). Importantly, basophils absence during ICU stay was positively correlated with the 28-day mortality of ICU patients and served as an independent predictor of ICU patients’ prognosis (OR 3.425, 95% CI 3.717–3.165, *P* < 0.001). Moreover, the association between critical illness progression, poor outcome, and basophils absence was verified in septic patients. Subsequent investigations revealed the positive relationship between basophils absence and immunosuppression, and suggested the potential of basophils-mediated immunity in predicting the 28-day mortality of ICU patients. Collectively, we identify basophils absence during ICU stay as a novel and unfavorable indicator for evaluating the prognosis of ICU patients and recognizing a branch of ICU patients potentially suitable for intensified treatment and immunoenhancement therapy.

## Introduction

The intensive care specialty has made important advances in the survival rates of patients with critical illnesses. However, critical illness with persistent morbidity is still complicated^1^. Therein, sepsis accounts for the majority of patients diagnosed in intensive care units (ICU) and is a primary challenge. Sepsis is an infection-related disease with approximately 20% of the deaths worldwide and is characterized by abnormal immune responses, dysregulated inflammation, and organ dysfunction^2,3^. At present, the prognostic assessment scales used for clinical practice in ICU are quite complicated, and it is meaningful to find a simple biomarker that can effectively evaluate the prognosis of ICU patients. It is also necessary for developing such a biomarker that facilitates clinicians’ decision-making when they are uncertain whether intensified treatments, such as the Early Management Bundle for Severe Sepsis/Septic Shock, should be used or not.

Recently, increasing evidence suggested that immunotherapy might be an attractive strategy for the treatment in ICU^4,5^. Both innate and adaptive immunity are dysregulated during the progression of critical illness^6,7^. Moreover, immune checkpoint inhibitors provided some indication of restored immune status and were well tolerated in patients with critical illness^8,9^. However, the immune system is not routinely monitored in ICU and the relationship among the change of specific immune-related indicators, immunity, and the prognosis of ICU patients is yet to be elucidated.

Several immune cells were shown to be associated with patients’ outcomes in ICU^10^. Absolute lymphocyte subsets levels and lymphocyte counts were downregulated in sepsis, and the proportion of Tregs was positively correlated with disease progression^11^. Moreover, myeloid subsets were also found to be associated with poor prognosis in patients with sepsis^12^. Furthermore, all the initial, maximum, mean, and difference values of a specific parameter from blood tests were reported to affect ICU patients’ prognosis^13–15^. However, the landscape of immune cells from blood tests in ICU and their roles in patient prognosis assessment have not been fully explored, and conducting further exploration regarding this will be interesting.

In the present investigation, we integrated two cohorts from the Medical Information Mart for Intensive Care IV (MIMIC-IV) to screen and validate an immune-related indicator in evaluating the outcome of ICU and septic patients, respectively. The relationships among the identified indicator (i.e., basophils status), clinicopathological parameters, and immunity of ICU patients were elucidated to measure the role of basophils status in predicting the prognosis of ICU patients. In addition, we also investigate the possibility of basophils as a promising biomarker for medical decision-making and the identification of ICU patients who may benefit from intensified treatment and immunoenhancement therapy.

## Methods

### Data sources

The MIMIC-IV database was utilized for data acquisition with ethics committee approval (certification No. 37303946). The MIMIC-IV was constructed as a large, single-center database comprising information on patients admitted to critical care units as described previously^16^. This retrospective study was carried out in accordance with Reporting of Studies Conducted using Observational Routinely Collected Health Data (RECORD)^17^. Since the investigation was performed by using the third-party anonymized public database, written informed consent from the patients/participants or their next of kin was not required according to national legislation and institutional requirements.

### Participants

Only the first admission of ICU patients with available blood tests was included for investigation, and patients younger than 18 years were excluded from the study. The medical records of demographics, nursing progress notes, laboratory results, medications, and international classification of diseases were used for data extraction from ICU and septic patients. Sepsis was diagnosed based on the sepsis-3 criteria. The mean, initial, maximum, and minimum values of the adopted immune cells from blood tests were extracted during ICU admission. The difference values of immune cells were defined as the maximum values the minus minimum values. Variables recorded more than once were calculated as the mean or median values during ICU stay.

### Primary and secondary outcomes

The primary outcome defined in this study was the 28-day mortality of patients after ICU admission. The secondary outcomes included 1-day, 7-day, and 14-day mortality of patients after ICU admission, norepinephrine and mechanical ventilation use during ICU stay, secondary acquired infections, ICU duration, as well as hospital duration.

### Statistical analysis

The data were analyzed by R software version 4.1.2 and SPSS 22.0. Demographic and admission information, comorbidities, vital signs, interventions, and laboratory results were extracted as variables. The definition of basophils absence (basophils-negative) was that the minimum values of basophils percentage detected from blood tests during ICU stay were equal to zero. The definition of basophils presence (basophils-positive) was that basophils (identified by the values of basophils percentage) were detectable from blood tests during ICU stay. For comparisons between groups, the continuous variables were expressed as the means (standard deviations) used for Student’s t-tests or medians (interquartile ranges) used for non-parametric tests. The categorical variables were expressed as total numbers (percentages) and analyzed by χ^2^ tests.

The univariate COX regression model was used to elucidate the relationship between blood immune cells and 28-day mortality of ICU patients. In the adjusted model, the odds ratio (OR) and 95% confidence interval (CI) were calculated by comparing the highest quartile with the lowest quartile as previously described^18^. The time-dependent receiver operating characteristic (ROC) curves were used to calculate the area under curves (AUCs). The log-rank test and Kaplan–Meier curves were utilized to detect the impact of basophils absence on patients’ survival. The best cut-off value of the minimum values of basophils percentage was determined with the aid of the survminer R package as previously described^19^. Univariate and multivariate logistic regression analyses were applied for exploring independent prognostic factors, and an odds ratio with a 95% CI was calculated. The differential analyses were conducted to estimate the difference in immune cells between the basophils-negative and basophils-positive groups.

The robustness of the findings in this study was assured and the covariates were adjusted by analyses with several models based on a previous report^20^: a logistic regression-based multivariate analyses model, a doubly robust model adjusting for unbalanced covariates or all covariates, a propensity score-based matching (PSM) model, a propensity score-based inverse probability weighting (IPW) model, and a subgroup analyses model. Multivariate analyses were duplicated after multiple imputation to avoid bias that might be caused by missing data. These models could be used to reveal the association and causal effects of exposure on an outcome.

All statistical tests were two-sided and a *P* value of < 0.05 was regarded as statistically significant.

### Ethics declarations

The author got access to the MIMIC-IV database with ethics committee approval from the Massachusetts Institute of Technology (Cambridge, MA) and Beth Israel Deaconess Medical Center (Boston, MA) and downloaded data for analyses (certification No. 37303946). Written informed consent from the patients/participants or their next of kin was not required to participate in this study in accordance with national legislation and institutional requirements. The procedures used in this study adhere to the tenets of the Declaration of Helsinki.

## Results

### Connections between immune cells and the prognosis of ICU patients

**Figure 1:**
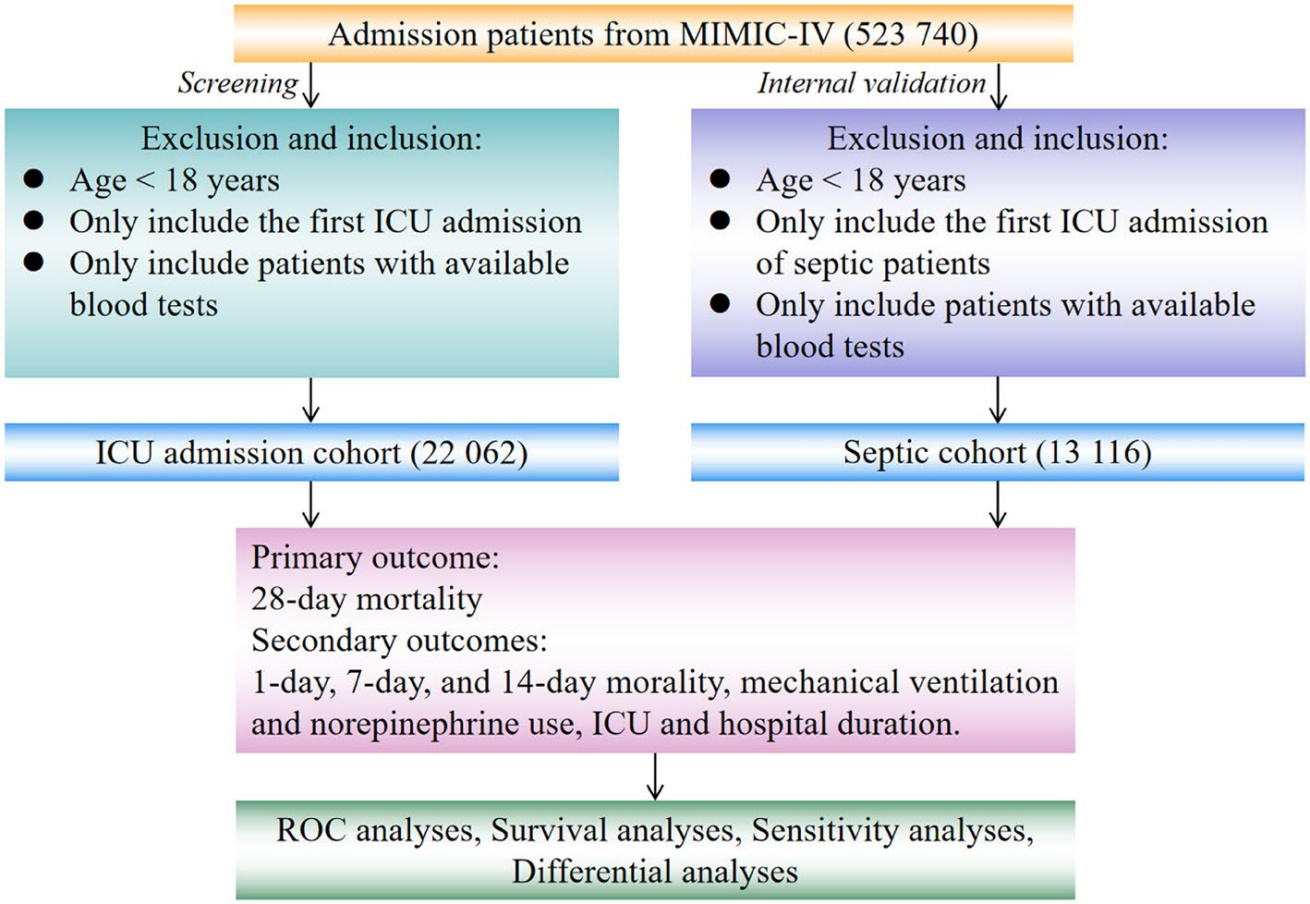
The flow diagram of this work. Illustration of the exclusion and inclusion criteria, outcomes, and statistical analyses adopted in this study.

To elucidate the role of immune cells in ICU patients’ prognosis, we conducted retrospective analyses in a large sample of ICU patients. After the initial search of MIMIC-IV database, we recognized 523,740 ICU admissions, and a total of 22,062 patients with the first admission and available blood tests were included in the study (Fig. 1). First, a total of 18 innate and adaptive immune cells-related parameters, including the cell counts and percentages of CD3-positive cells, CD4-positive cells, CD8-positive cells, basophils, eosinophils, lymphocytes, monocytes, and neutrophils, as well as the cell counts of immature granulocytes and plasma cells, were adopted for survival analyses with a univariate COX regression model to elucidate the relationship between blood immune cells and 28-day mortality of ICU patients. The cell counts and percentages of basophils, eosinophils, and lymphocytes predicted a good prognosis for ICU patients, whereas the cell counts of immature granulocytes, monocytes, neutrophils, and plasma cells conferred a poor prognosis for ICU patients (Table 1). Therein, basophils harbored a superior role in predicting ICU patients’ prognosis compared with other blood immune cells (basophils percentage, adjusted OR 0.653, 95% CI 0.628–0.679). Next, we focused on basophils for further exploration (Fig. S1a,b). In terms of 28-day mortality of ICU patients, the minimum values of basophils percentage detected during ICU stay were superior to the maximum, mean, or the difference values of basophils percentages and counts in predicting ICU patients prognosis (Table S1), providing the basis for revealing the prognostic role of the minimum values of basophils percentage in further investigations. These findings suggested the predictive role of immune cells, especially for basophils, in the prognosis assessment of ICU patients.

**Table 1:** The relationship between blood immune cells and 28-day mortality of ICU patients.

Immune cells	OR (95% CI)	*P* value	Adjusted OR (95% CI)	*P* value
Basophils count	0.013 (0.001, 0.118)	< 0.001	0.723 (0.680, 0.768)	< 0.001
CD3-positive cells count	1 (0.999, 1.001)	0.448	0.728 (0.449, 1.180)	0.198
CD4-positive cells count	1 (0.997, 1.002)	0.660	0.834 (0.502, 1.385)	0.482
CD8-positive cells count	0.999 (0.997, 1.001)	0.240	0.628 (0.361, 1.091)	0.099
Eosinophils count	0.686 (0.471, 0.999)	0.049	0.784 (0.736, 0.835)	< 0.001
Immature granulocytes count	1.618 (1.446, 1.810)	< 0.001	1.405 (1.294, 1.526)	< 0.001
Lymphocytes count	0.999 (0.999, 1)	0.047	0.716 (0.669, 0.766)	< 0.001
Monocytes count	1.155 (1.071, 1.245)	< 0.001	1.132 (1.062, 1.206)	< 0.001
Neutrophils count	1.042 (1.034, 1.051)	< 0.001	1.284 (1.205, 1.369)	< 0.001
Plasma cells count	1.783 (1.045, 3.044)	0.034	–	–
Basophils percentage	0.192 (0.156, 0.236)	< 0.001	0.653 (0.628, 0.679)	< 0.001
CD3-positive cells percentage	1.001 (0.970, 1.033)	0.959	1.008 (0.977, 1.040)	0.625
CD4-positive cells percentage	1.023 (0.994, 1.053)	0.116	1.029 (0.993, 1.065)	0.113
CD8-positive cells percentage	0.979 (0.952, 1.006)	0.122	0.969 (0.935, 1.004)	0.084
Eosinophils percentage	0.846 (0.814, 0.879)	< 0.001	0.682 (0.651, 0.714)	< 0.001
Lymphocytes percentage	0.969 (0.962, 0.974)	< 0.001	0.673 (0.644, 0.704)	< 0.001
Monocytes percentage	1.006 (0.995, 1.018)	0.286	0.976 (0.937, 1.017)	0.241
Neutrophils percentage	0.999 (0.996, 1.003)	0.676	1.174 (1.127, 1.223)	< 0.001

*ICU* intensive care unit, *OR* odds ratio, *CI* confidence interval.

### Demographic data and baseline characteristics

**Figure 2:**
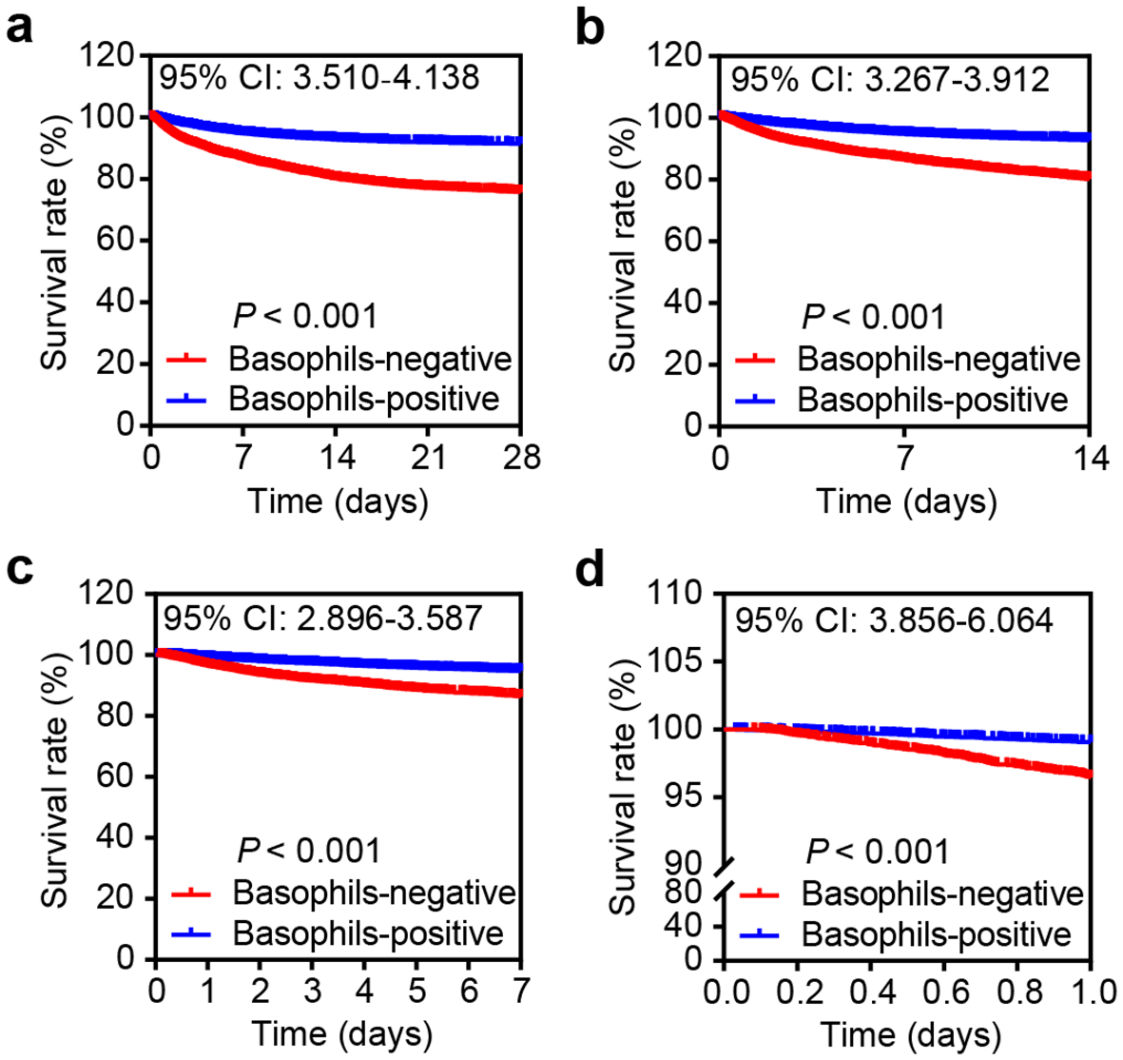
Basophils absence predicts poor prognosis of ICU patients. **(a–d)** Patients in the basophils-negative group had poor 28-day **(a)**, 14-day **(b)**, 7-day **(c)**, and 1-day **(d)** survival rates in comparison to ICU patients in the basophils-positive group.

Subsequently, we focused on the minimum values of basophils percentage for further investigation (Fig. S1c,d). To further investigate the role of basophils in ICU patients, the survival analysis was performed to elucidate the best cut-off value. The best cut-off value of the minimum values of basophils percentage determined by the survival analysis was 0 in the 22,062 ICU patients. Therefore, a total of 6344 patients (the minimum values of basophils percentage = 0) were divided into basophils-negative (basophils absence) group, and the remaining 15,718 samples (the minimum values of basophils percentage > 0) were regarded as the basophils-positive (basophils presence) group. The ICU patients in the basophils-negative group had a poor prognosis in comparison to those in the basophils-positive group (overall 28-day mortality rate: 24.23% vs 8.54%; overall 14-day mortality rate: 19.81% vs 7.20%; overall 7-day mortality rate: 13.54% vs 5.21%; overall first-day mortality rate: 3.55% vs 0.94%) (Fig. 2a–d). Moreover, Table 2 showed the differential distribution of clinicopathologic characteristics of ICU patients. The percentages of missing data in the variables of interest were shown in Table S2. Patients in the basophils-negative groups had more severe illness (i.e., higher SOFA score, higher usage of renal replacement therapy, mechanical ventilation, and noradrenaline as well as more prominent abnormalities in laboratory biochemical tests and vital signs) compared to those in the basophils-positive group. In addition, the presence of cancer and liver diseases was more common in the basophils-negative group with more patients harboring coronary and cerebrovascular diseases in the basophils-positive group.

**Table 2:** Baseline characteristics of ICU patients between basophils-negative and basophils-positive groups.

Variables	Basophils-negative	Basophils-positive	*P* value
Age (years)	64 (52, 75)	66 (56, 75)	0.008
Male, n (%)	3452 (54.4)	9351 (59.5)	< 0.001
BMI (kg/m^2^)	28.07 (24.31, 33.18)	28.57 (24.99, 33.15)	0.006
Admission type, n (%)			< 0.001
Elective	1091 (17.2)	4621 (29.4)	
Emergency/urgent	5253 (82.8)	11,097 (70.6)	
SOFA score	9 (6, 12)	6 (4, 8)	< 0.001
Renal replacement therapy	921 (14.5)	775 (4.9)	< 0.001
Noradrenaline use	2831 (44.6)	2930 (18.6)	< 0.001
Mechanical ventilation	3637 (57.3)	7380 (47)	< 0.001
Service unit (MICU%)	3847 (60.6)	5501 (35)	< 0.001
Laboratory tests			
Bicarbonate	23 (20.25, 25.96)	23.9 (22, 25.75)	< 0.001
Hemoglobin	9.35 (8.45, 10.46)	9.92 (8.89, 11.10)	< 0.001
Lactate	1.92 (1.37, 2.79)	1.78 (1.35, 2.34)	< 0.001
PCO2	40 (36, 44.5)	40.6 (37.45, 44.17)	< 0.001
Ph	7.38 (7.33, 7,41)	7.39 (7.36, 7.42)	< 0.001
Urea-nitrogen	29.11 (17.66, 46.50)	17.75 (13.2, 27)	< 0.001
White blood cell	13.23 (9.59, 18.12)	12.22 (9.7, 15.1)	< 0.001
Potassium	4.07 (3.84, 4.36)	4.21 (3.95, 4.48)	< 0.001
Sodium	139 (136.26, 141.83)	138.17 (136, 140.56)	0.215
Comorbidities, (n%)			
Hypertension	1569 (24.7)	3887 (24.7)	0.997
CPD	1587 (25)	3813 (24.3)	0.237
Coronary disease	1349 (21.3)	5931 (37.7)	< 0.001
CHF	1665 (26.2)	4310 (27.4)	0.075
Cancer	1618 (25.5)	1737 (11.1)	< 0.001
Liver disease	1229 (19.4)	1714 (10.9)	< 0.001
Renal disease	1273 (20.1)	3038 (19.3)	0.211
Cerebrovascular disease	679 (10.7)	2201 (14)	< 0.001
Vital signs			
MAP (mmHg)	56 (48, 78.5)	59 (53, 81)	< 0.001
Heart rate (bpm)	112 (97, 129)	98 (79, 111)	< 0.001
Temperature (°C)	36.5 (35.78, 37.83)	36.39 (35.78, 37.28)	< 0.001
Respiratory rate (bpm)	29 (25, 34)	27 (23, 31)	< 0.001

*ICU* intensive care unit, *BMI* body mass index, *MICU* medical intensive care unit, *SOFA* sequential organ failure assessment, *CPD* chronic pulmonary disease, *CHF* congestive heart failure, *MAP* mean arterial pressure.

### Associations between basophils absence and the outcome of ICU patients

To verify the relationship between basophils absence and ICU patients’ outcomes, univariate and multivariate logistic regression analyses were performed and demonstrated that basophils presence during ICU stay predicted superior 28-day survival of ICU patients (OR 0.292, 95% CI 0.269–0.316) (Table S3). Moreover, age, admission type, SOFA score, renal replacement therapy, norepinephrine use, mechanical ventilation use, hemoglobin, lactate, PCO2, Ph, Urea-nitrogen, white blood cell, sodium, congestive heart failure, cancer, liver disease, cerebrovascular disease, and heart rate were revealed as independent unfavorable indicators, and male, BMI, bicarbonate, coronary disease, and temperature were classified as independent favorable indicators in predicting ICU patients prognosis. Interestingly, the basophils presence served as an independent and powerful protective factor in the prediction of ICU patients’ prognosis among all the parameters adopted for analyses. In the subgroup analyses of ICU patients, the negative association between basophils presence and 28-day mortality remained significant (Fig. S2), further suggesting the positive association between basophils absence and poor outcome of ICU patients. Then, multivariate after multiple imputation, a doubly robust model adjusting for unbalanced covariates or all covariates, a PSM model, and a propensity score-based IPW model were used to verify the association between basophils absence and 28-day mortality of ICU patients. Therein, PSM was performed by a 1:1 matching algorithm, and 1057 patients in the basophils-negative group were matched to 1057 patients in the basophils-positive group with the only differential distribution of urea-nitrogen between these two groups (Table S4, Fig. S3). As shown in Table 3, the analyses with all 5 estimation models confirmed that ICU patients with basophils absence had poor 28-day survival. To further explore the role of basophils in ICU patients, we measured several secondary outcomes to elucidate potential factors that correlated with the basophils status. PSM was then performed by a 1:1 matching algorithm and the differential analyses revealed that the rate of norepinephrine and mechanical ventilation use of patients during ICU stay was higher in the basophils-negative group compared to those in the basophils-positive group. Moreover, the ICU duration and hospital duration of basophils-negative ICU patients were longer than those of basophils-positive ICU patients (Table S5).

**Table 3:** Primary outcome analyses with 5 different models elucidating the role of basophils absence in ICU patients.

Methods	Odds ratio (95% CI)	*P* value	Adjusted odds ratio (95% CI)	Adjusted *P* value
Multivariate after multiple imputation	1.238 (1.110, 1.379)	< 0.001	1.883 (1.406, 2.387)	< 0.001
Doubly robust with all covariates	1.312 (1.130, 1.522)	< 0.001	1.631 (1.276, 2.088)	< 0.001
Doubly robust with unbalanced covariates	1.299 (1.121, 1.506)	0.001	1.621 (1.269, 2.070)	< 0.001
Propensity score matching	1.427 (1.125, 1.808)	0.003	1.212 (1.140, 1.321)	< 0.001
Propensity score IPW	1.104 (1.027, 1.186)	0.007	1.745 (1.404, 2.169)	< 0.001

*ICU* intensive care unit; *IPW* inverse probability weighting.

Collectively, these findings suggest the predictive role of basophils absence in the prognostic evaluation of ICU patients.

### Internal validation of the role of basophils absence in ICU patients with sepsis

The internal validation of the relationship between basophils absence and the patients’ outcome was then conducted in ICU patients with sepsis. Therein, the proportion of septic patients was 78.66% and 51.70% in the basophils-negative and basophils-positive groups of ICU patients, respectively. The primary diagnosis of ICU admission was shown in Table S6. First, basophils absence was shown to confer a poor prognosis at 1, 7, 14, and 28 days for the septic patients (Fig. S4a–d). Then, the relationship between basophils absence and illness severity was detected, and septic patients with basophils absence were characterized by increased severity of illness (Table S7). Table S8 presented the differential distribution of microbiological characteristics between basophils-negative and basophils-positive groups. Moreover, univariate and multivariate logistic regression analyses also suggested that septic patients with basophils presence had a good prognosis (OR 0.386, 95% CI 0.352–0.423; *P* < 0.001) (Table S9), indicating that basophils absence served as an independent and adverse prognostic factor in septic patients. In addition, the analyses with all 5 estimation models also verified the positive association between basophils absence and 28-day mortality in ICU septic patients (Table S10). Moreover, the rates of norepinephrine use, mechanical ventilation use, and secondary acquired infections, as well as the ICU and hospital duration of basophils-negative septic patients, were higher than those of basophils-positive septic patients (Table S11). These findings collectively validated the predictive role of basophils absence in the prognostic evaluation of ICU patients with sepsis.

### Connections between basophils absence and the immunity of ICU and septic patients

**Figure 3:**
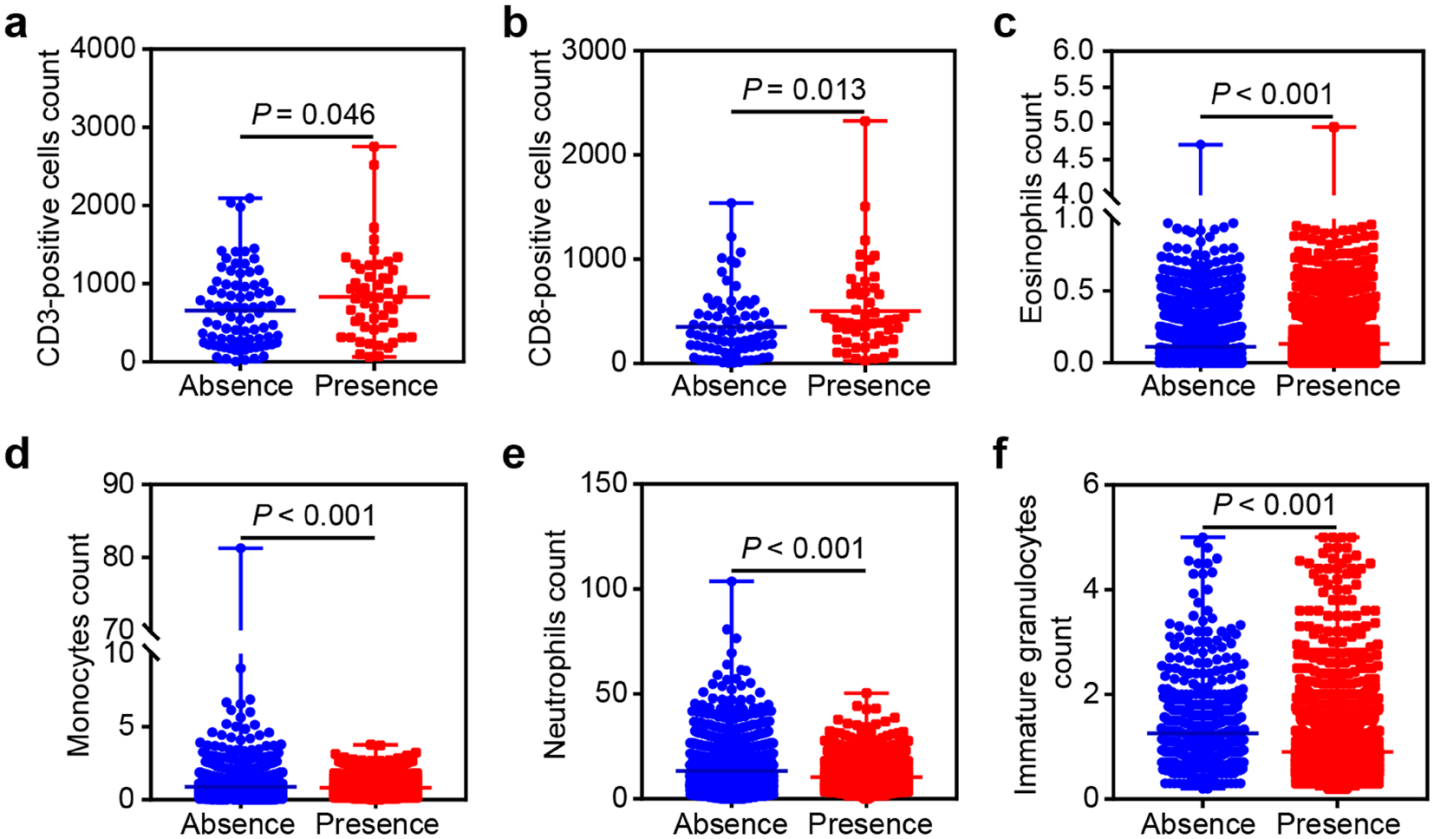
The relationship between basophils status and immunity of ICU patients. The differential distribution of the cell counts of CD3-positive cells **(a)**, CD8-positive cells **(b)**, eosinophils **(c)**, monocytes **(d)**, neutrophils **(e)**, and immature granulocytes **(f)** between the basophils absence and basophils presence groups.

Basophils were identified as important contributors to memory immune responses^21^. Next, the relationship between basophils absence and the immunity of ICU patients was further investigated, and the aforementioned innate and adaptive immune cells were applied for analyses. The data revealed the correlations between immune cells and the 28-day mortality of ICU patients (Table 1). Consistently, the counts of CD3-positive cells, CD8-positive cells, and eosinophils were elevated in the basophils presence group compared to those in the basophils absence group. However, basophils absence was associated with increased monocytes, neutrophils, and immature granulocytes counts (Fig. 3a–f, Fig. S5a–f). These data indicated the potential role of basophils absence-related immunity in predicting the prognosis of ICU and septic patients.

## Discussion

Nowadays, immunosuppression in critical patients has drawn more and more attention^22^. Although several scoring systems have been applied as prognostic assessment scales for ICU in the past few decades^23^, almost none of them emphasized the importance of immunity in ICU patients and incorporated specific immune-related indicators into the scales. In this investigation, we adopted innate and adaptive immune cells from blood tests for analyses and found that basophils had a superior role in the prediction of ICU patients’ prognosis. Furthermore, we suggested that basophils absence was correlated with poor patients’ prognosis, and basophils status (i.e., absence or presence) of patients during ICU stay served as an independent prognostic indicator in both ICU and septic patients. Moreover, basophils absence indicated an immunosuppression status in ICU and septic patients, and the potential application of basophils status for the prognosis assessment of ICU and septic patients in clinical practice might be concerned.

Previous studies showed that the decrease of basophils in severe COVID-19 patients and the decrease of basophils were associated with increased mortality in COVID-19 patients admitted to ICU^24^. Moreover, basophils percentage was demonstrated to be independently correlated with ICU morality^25^. In sepsis, basophils could strengthen the innate immune response to enhance survival in a mouse model^26^ and were shown to be elevated in surviving patients with sepsis^27^. When classifying immune-associated cells from peripheral blood in our investigation, we found that both increased basophils count and percentage showed a protective role in ICU patients. Moreover, basophils were shown to be the most powerful biomarker for prognostic assessment among the 18 adopted innate and adaptive immune cells. Interestingly, our results further showed that basophils absence was correlated with poor prognosis in ICU and septic patients. For the first time, we proposed that basophils status (i.e., absence or presence) during ICU stay served as an independent biomarker in predicting ICU and septic patients’ prognosis. Interestingly, we applied several methods, including logistic regression analyses, doubly robust analyses, propensity score IPW, propensity score matching, multivariate regression analyses after multiple imputation, and subgroup analyses, to verify basophils status as an independent prognostic indicator after adjusting for multiple clinicopathological characteristics in ICU and septic patients. Although the above-mentioned reports suggested the association between basophils and patients’ prognosis, no existing evaluation system embedded basophils as a parameter. Some of the applied prognostic assessment scales, such as SOFA, SIRS, SAPS II, OASIS, LODS, and APACHE III, included circulating leukocytes as a parameter for scoring^28–30^. Here, we deemed that it is time to consider basophils status for prognostic evaluation in ICU and septic patients since the basophils status was easily obtained from blood routine tests and had a superior role in predicting patients’ prognosis compared to circulating leukocytes based on our univariate and multivariate logistic regression analyses. Moreover, basophils absence could also distinguish ICU patients with a high risk of norepinephrine use, mechanical ventilation use, long ICU duration, and long hospital duration from those with a low risk, and might function as a biomarker for the selection of ICU patients receiving intensified therapy before disease progression. In addition, the neutrophil-to-lymphocyte ratio was a well-known prognostic indicator that was independently associated with mortality in the general population and in several specific disease subsets, such as sepsis^31^. Here, we found that basophils absence had a comparable role in predicting the prognosis compared to the neutrophil-to-lymphocyte ratio in septic patients (Tables S12, S13), further indicating basophils absence as a promising biomarker.

The immune system exerts a crucial role in critical illness and has become a research hotspot^32^. Immunosuppression is an outstanding characteristic of sepsis, and reversing sepsis-induced immunosuppression might be an alternative strategy for sepsis treatment^33^. Therefore, we extracted innate and adaptive immune cells from blood tests to clarify the relationship between immune responses and critical illness in the present study. Low CD3-positive cells might be correlated with the mortality of ICU patients^34^. CD8-positive cells exhaustion was shown to be associated with a worse survival rate in septic patients^35^. Low eosinophils count predicted poor outcome in ICU patients^36^. Elevated monocytes counts were independently associated with mortality in severe sepsis^37^. High neutrophils served as a predictor of death in ICU patients^38^. Increased circulating immature granulocytes were related to worsening sepsis at the acute phase^39^. These reports indicated the protective roles of CD3-positive cells, CD4-positive cells, and eosinophils as well as the promotive roles of neutrophils, monocytes, and immature granulocytes in critical illness. Consistent with previous findings, we confirmed the positive correlation between basophils absence and the cell counts of neutrophils, monocytes, and immature granulocytes as well as the negative correlation between basophils absence and the cell counts of CD3-positive cells, CD4-positive cells, and eosinophils, further suggesting the tight connection between basophils absence and immunosuppression. Moreover, immunoenhancement therapy strategies for ICU and septic patients with basophils absence might be considered.

Some limitations should be noticed in the present work. First, several factors affect the parameters of blood tests, and clarifying the causal relationship between basophils absence and the prognosis of ICU patients by adjusting for all these factors, such as therapeutic interventions, is somehow difficult for a retrospective observational study. Second, sepsis guidelines were updated during the period that septic patients encompassed the MIMIC IV databases, which might be a confounding factor impacting the current application of our findings. Finally, this was a retrospective cohort study, and a randomized study in the future is needed to validate the role of basophils status in the prognostic evaluation of ICU patients.

The application of the real-world data obtained from MIMIC-IV could somehow sufficiently assess the clinical effectiveness of specific tests. Our work portrays the link among basophils absence, immunity, and prognosis of ICU patients. Moreover, basophils absence may be used as a promising biomarker for medical decision-making and individualized treatment, especially for the identification of ICU and septic patients who may benefit from intensified treatment and immunoenhancement therapy, thus effectively ameliorating patients’ prognosis.

### Supplementary Information

Supplementary Information.

## Data Availability

The datasets used and/or analyzed during the current study are available from the corresponding author on reasonable request.
