# Supplementary material for: Basophils absence predicts poor prognosis and indicates immunosuppression of patients in intensive care units
Source: Sci Rep. 2023 Oct 28;13:18533. doi: 10.1038/s41598-023-45865-y (PMC10613308; doi:10.1038/s41598-023-45865-y)
Supplement: Supplementary file 1 — Supplementary Information. [file 41598_2023_45865_MOESM1_ESM.doc]

**Basophils absence predicts poor prognosis and indicates immunosuppression of patients in intensive care units**

Xiao Chen^1, 2, #^, Xiaofeng Zhu^3, #^, Huichang Zhuo^1, 2^, Jiandong Lin^1, 2^, **^*^**, Xian Lin^4^, **^*^**

^1^Department of Intensive Care Unit and The Clinical Key Specialty of Fujian Province, First Affiliated Hospital of Fujian Medical University, Fuzhou, Fujian, China;

^2^Department of Intensive Care Unit, National Regional Medical Center, Binhai Campus of the First Affiliated Hospital, Fujian Medical University, Fuzhou, Fujian, China;

^3^Department of Oral Maxillo-Facial Surgery, The First Affiliated Hospital of Fujian Medical University, Fuzhou, Fujian, China;

^4^Shenzhen Key Laboratory of Immunity and Inflammatory Diseases, Peking University Shenzhen Hospital, Shenzhen Peking University-The Hong Kong University of Science and Technology Medical Center, Shenzhen, Guangdong, China.

^#^These authors have contributed equally to this work.

*** Correspondence:**

**Jiandong Lin**, Department of Intensive Care Unit, First Affiliated Hospital of Fujian Medical University, 20 Chazhong Road, Taijiang District, Fuzhou 350001, Fujian, China. Telephone: +86-0591-87981982. Fax: +86-0591-87983333. Email: linjd01680067@sina.com

**Xian Lin**, Shenzhen Key Laboratory of Immunity and Inflammatory Diseases, Peking University Shenzhen Hospital, Shenzhen Peking University-The Hong Kong University of Science and Technology Medical Center, 1120 Lianhua Road, Futian District, Shenzhen 518036, Guangdong, China. Telephone: +86-15626040245. Fax: +86-0755-83061340. Email: linxiangabriel@fjmu.edu.cn

Xiao Chen, Email: chenxiao350001@163.com; Xiaofeng Zhu, Email: dentzxf@163.com or zhuxiaofengfjmu@163.com; Huichang Zhuo, Email: zhc41266371@163.com

**This file includes:** Fig. S1-5 and Table S1-13.

**Supplementary Figures and Figure legends**

**
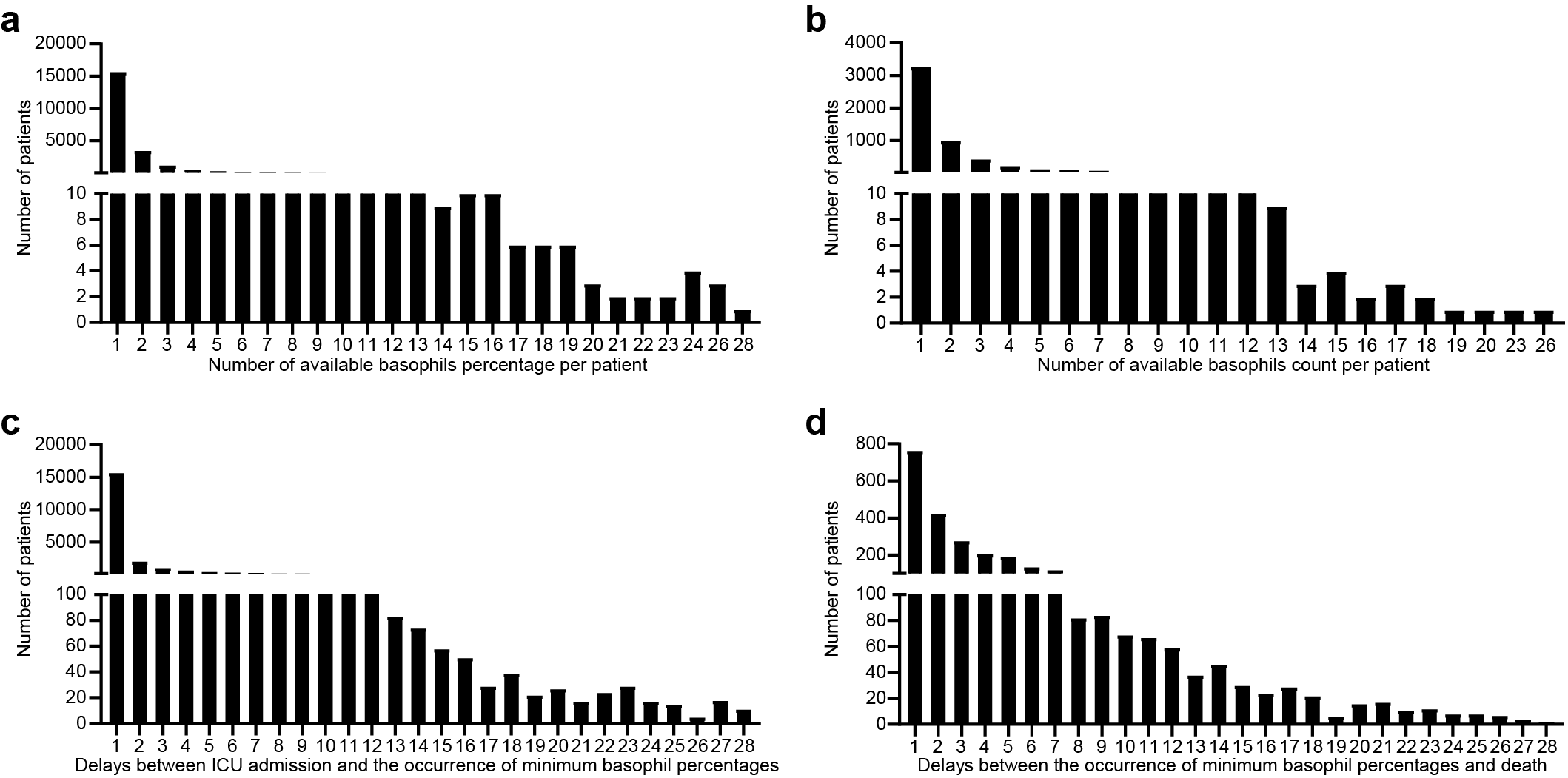
**

**Fig. S1** The global description of basophils percentages and counts. **(a, b)** Distribution of the number of basophils percentages **(a)** and counts **(b)** tested during patients’ ICU stay. **(c)** Delays between ICU admission and the occurrence of minimum basophil percentages tested during patients’ ICU stay. **(d)** Delays between death and the occurrence of minimum basophil percentages tested during patients’ ICU stay.

**
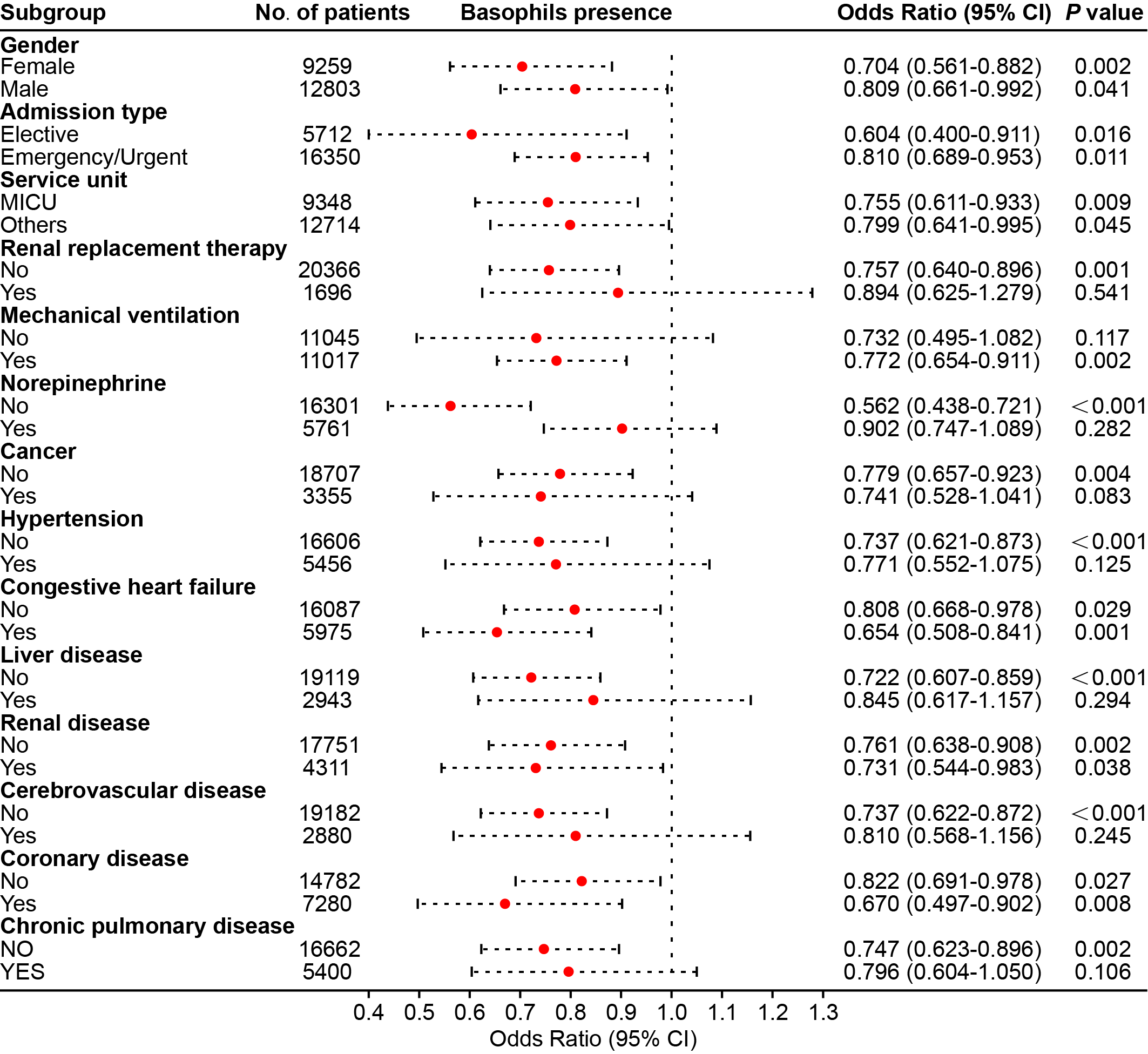
**

**Fig. S2** Subgroup analyses of the association between basophils absence and 28-day mortality in ICU patients. Subgroup analyses were performed to confirm basophils status as an independent prognostic indicator.


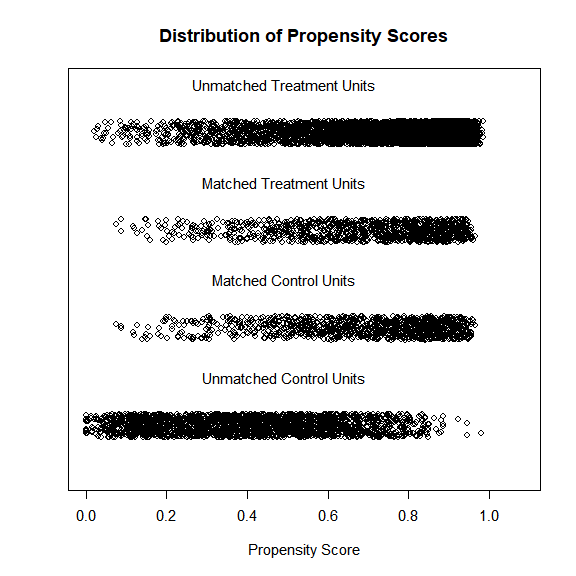

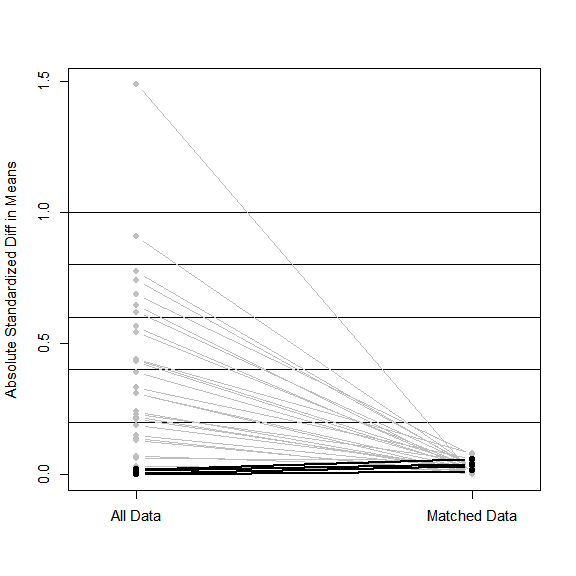


**Fig. S3** A balanced distribution of baseline characteristics between basophils-negative and basophils-positive groups after propensity score matching. Only differential distribution of urea-nitrogen was detected after propensity score matching.

**
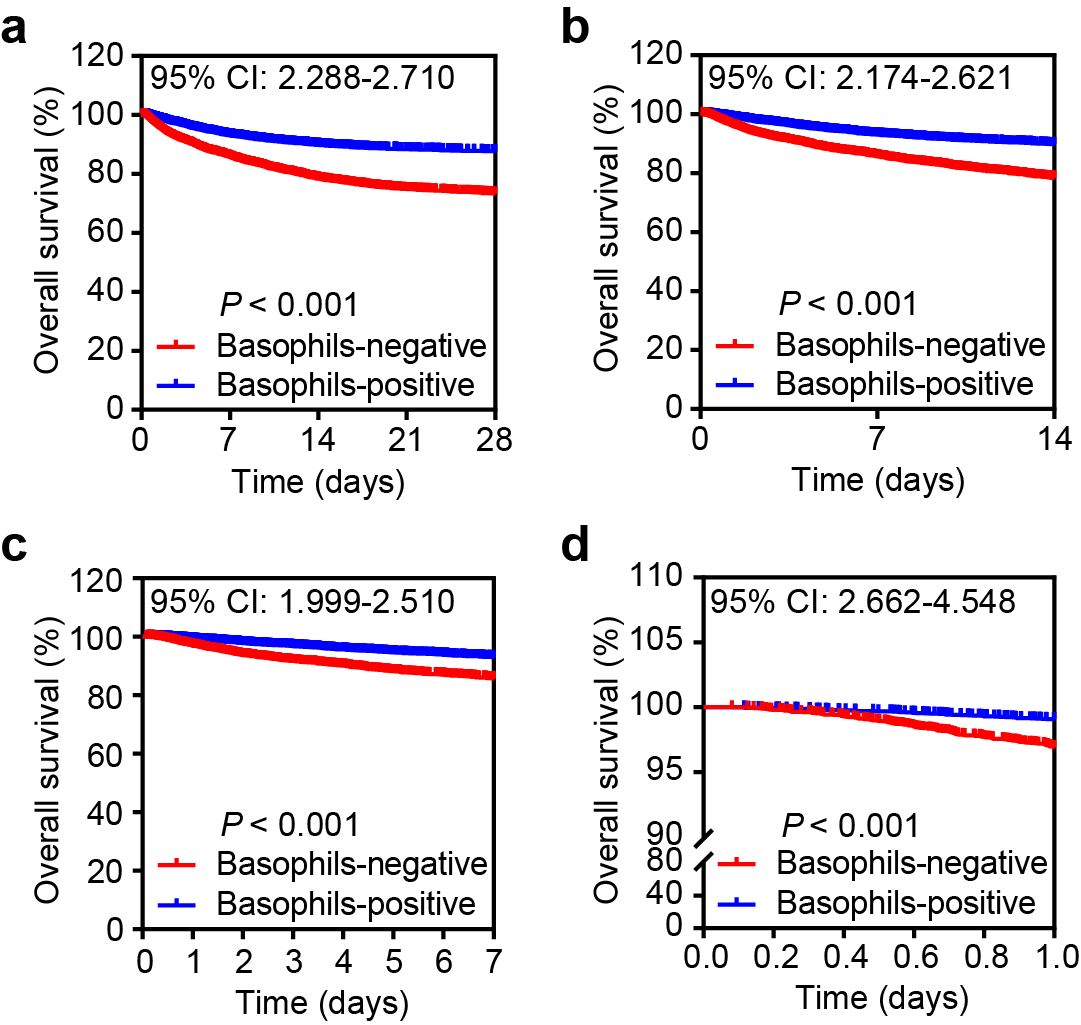
**

**Fig. S4** Basophils absence predicts poor prognosis of septic patients. **(a-d)** Patients in the basophils-negative group had poor 28-day **(a)**, 14-day **(b)**, 7-day **(c)**, and 1-day **(d)** survival rates in comparison to septic patients in the basophils-positive group.

**
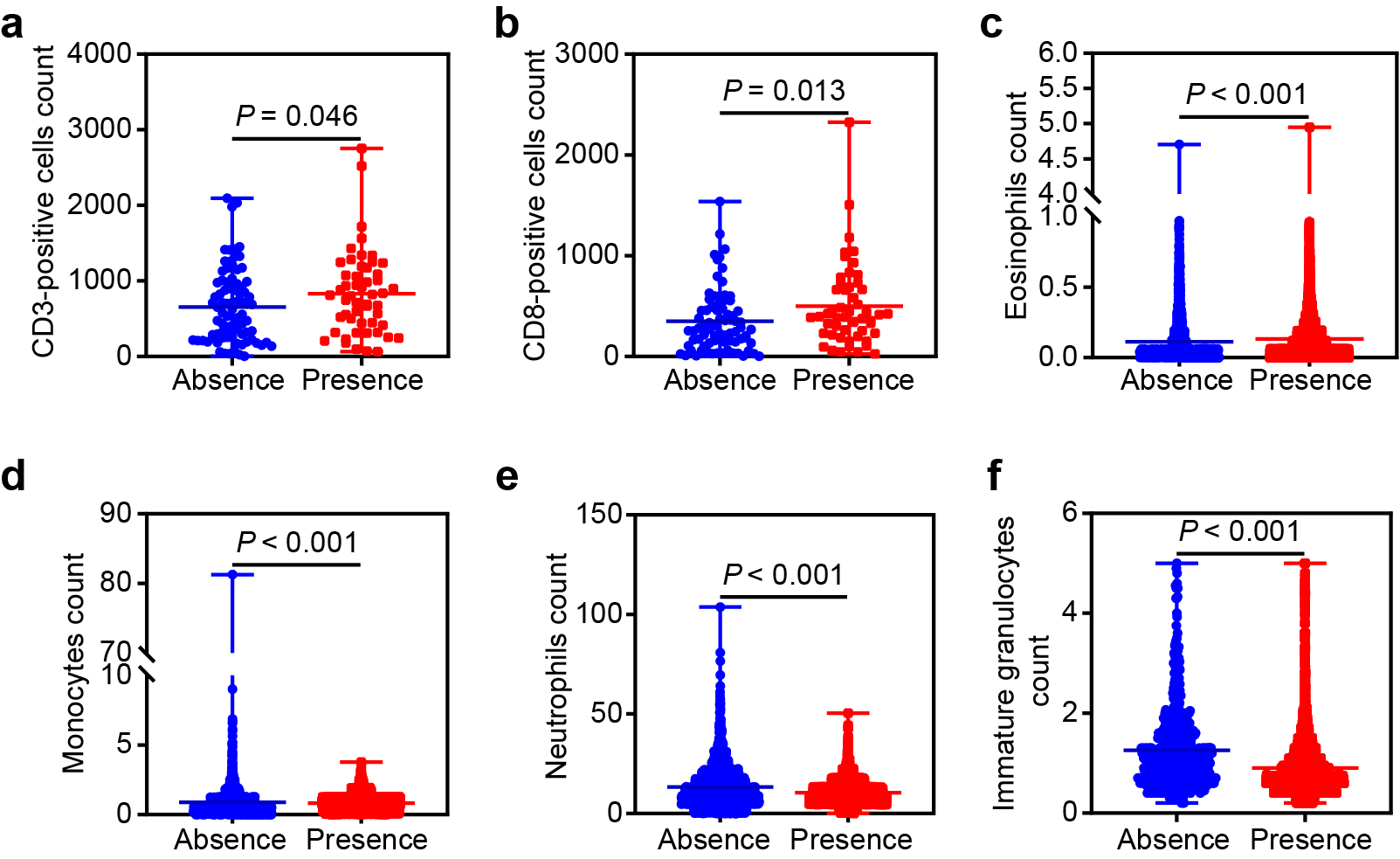
**

**Fig. S5** The relationship between basophils status and immunity of septic patients. The differential distribution of the cell counts of CD3-positive cells **(a)**, CD8-positive cells **(b)**, eosinophils **(c)**, monocytes **(d)**, neutrophils **(e)**, and immature granulocytes **(f)** between the basophils absence and basophils presence groups.

**Supplementary tables**

**Table S1.** The area under curves of basophils in predicting 28-day mortality of ICU patients.

| Basophils counts and percentages | Area under curves (95% CI) |
| --- | --- |
| Basophils initial percentage | 0.625 (0.613, 0.637) |
| Basophils maximum percentage | 0.594 (0.582, 0.606) |
| Basophils mean percentage | 0.631 (0.619, 0.642) |
| Basophils minimum percentage | 0.667 (0.656, 0.678) |
| Basophils difference percentage | 0.444 (0.433, 0.456) |
| Basophils initial count | 0.587 (0.566, 0.608) |
| Basophils maximum count | 0.565 (0.543, 0.586) |
| Basophils mean count | 0.596 (0.576, 0.617) |
| Basophils minimum count | 0.633 (0.614, 0.653) |
| Basophils difference count | 0.45 (0.430, 0.471) |

ICU: Intensive care unit

**Table S2.** The percentages of missing data in the variables of interest.

| Variables | MIMIC-IV (n = 22062) |
| --- | --- |
| Age (years) | 0% |
| Sex | 0% |
| BMI (kg/m^2^) | 38.41% |
| Admission type | 0% |
| SOFA score | 0% |
| Renal replacement therapy | 0% |
| Noradrenaline use | 0% |
| Mechanical ventilation | 0% |
| Service unit | 0% |
| Laboratory tests |  |
| Bicarbonate | 0.15% |
| Hemoglobin | 0.01% |
| Lactate | 23.96% |
| PCO2 | 26.10% |
| Ph | 23.86% |
| Urea-nitrogen | 0.17% |
| White blood cell | 0% |
| Potassium | 0.12% |
| Sodium | 0.15% |
| Comorbidities |  |
| Hypertension | 0% |
| CPD | 0% |
| Coronary disease | 0% |
| CHF | 0% |
| Cancer | 0% |
| Liver disease | 0% |
| Renal disease | 0% |
| Cerebrovascular disease | 0% |
| Vital signs |  |
| MAP (mmHg) | 0.22% |
| Heart rate (bpm) | 0.21% |
| Temperature (℃) | 4.05% |
| Respiratory rate (bpm) | 0.25% |

BMI: Body mass index; SOFA: Sequential organ failure assessment; CPD: Chronic pulmonary disease; CHF: Congestive heart failure; MAP: Mean arterial pressure

**Table S3.** Univariate and multivariate models assessing the impact of basophils status and other clinical factors on 28-day mortality in ICU patients.

| Variables | Univariate model | | Multivariate model | |
| --- | --- | --- | --- | --- |
|  | Odds Ratio  (95% CI) | *P* value | Odds Ratio  (95% CI) | *P* value |
| Basophils presence | 0.292 (0.269, 0.316) | ＜0.001 | 0.762 (0.656, 0.886) | ＜0.001 |
| Age | 1.019 (1.016, 1.021) | ＜0.001 | 1.025 (1.019, 1.030) | ＜0.001 |
| Gender/male | 0.854 (0.790, 0.924) | ＜0.001 | 0.744 (0.645, 0.859) | ＜0.001 |
| BMI | 0.988 (0.981, 0.995) | 0.001 | 0.977 (0.968, 0.986) | ＜0.001 |
| Admission type | 2.515 (2.248, 2.814) | ＜0.001 | 2.064 (1.719, 2.478) | ＜0.001 |
| SOFA score | 1.282 (1.269, 1.295) | ＜0.001 | 1.023 (1, 1.046) | 0.049 |
| Renal replacement therapy | 4.034 (3.615, 4.500) | ＜0.001 | 1.383 (1.136, 1.683) | 0.001 |
| Noradrenaline use | 6.218 (5.723, 6.756) | ＜0.001 | 2.300 (1.941, 2.724) | ＜0.001 |
| Mechanical ventilation | 2.718 (2.496, 2.959) | ＜0.001 | 1.766 (1.444, 2.161) | ＜0.001 |
| Service unit (MICU%) | 1.904 (1.759, 2.061) | ＜0.001 | 1.073 (0.919, 1.254) | 0.371 |
| Laboratory tests |  |  |  |  |
| Bicarbonate | 0.838 (0.829, 0.847) | ＜0.001 | 0.802 (0.772, 0.833) | ＜0.001 |
| Hemoglobin | 0.873 (0.854, 0.893) | ＜0.001 | 1.096 (1.048, 1.145) | ＜0.001 |
| Lactate | 1.797 (1.739, 1.857) | ＜0.001 | 1.452 (1.375, 1.534) | ＜0.001 |
| PCO2 | 1.008 (1.004, 1.013) | ＜0.001 | 1.121 (1.101, 1.142) | ＜0.001 |
| Ph | 0 | ＜0.001 | 96.001 (11.299, 815.626) | ＜0.001 |
| Urea-nitrogen | 1.029 (1.027, 1.031) | ＜0.001 | 1.016 (1.013, 1.020) | ＜0.001 |
| White blood cell | 1.027 (1.023, 1.031) | ＜0.001 | 1.010 (1.003, 1.018) | 0.006 |
| Potassium | 1.837 (1.699, 1.986) | ＜0.001 | 0.934 (0.783, 1.114) | 0.447 |
| Sodium | 1.034 (1.025, 1.043) | ＜0.001 | 1.023 (1.007, 1.039) | 0.004 |
| Comorbidities |  |  |  |  |
| Hypertension | 0.896 (0.817, 0.983) | 0.021 | 1.056 (0.885, 1.261) | 0.546 |
| CPD | 1.230 (1.126, 1.343) | ＜0.001 | 1.044 (0.893, 1.221) | 0.590 |
| Coronary disease | 0.661 (0.604, 0.722) | ＜0.001 | 0.675 (0.572, 0.796) | ＜0.001 |
| CHF | 1.345 (1.236, 1.463) | ＜0.001 | 1.204 (1.030, 1.407) | 0.020 |
| Cancer | 2.329 (2.123, 2.554) | ＜0.001 | 2.070 (1.725, 2.483) | ＜0.001 |
| Liver disease | 2.660 (2.420, 2.924) | ＜0.001 | 1.59 (1.328, 1.902) | ＜0.001 |
| Renal disease | 1.483 (1.354, 1.625) | ＜0.001 | 0.944 (0.785, 1.134) | 0.536 |
| Cerebrovascular disease | 1.567 (1.412, 1.739) | ＜0.001 | 2.848 (2.376, 3.414) | ＜0.001 |
| Vital signs |  |  |  |  |
| MAP (mmHg) | 0.988 (0.986, 0.989) | ＜0.001 | 1 (0.998, 1.002) | 0.989 |
| Heart rate (bpm) | 1.016 (1.014, 1.017) | ＜0.001 | 1.006 (1.003, 1.009) | ＜0.001 |
| Temperature (℃) | 0.816 (0.790, 0.842) | ＜0.001 | 0.924 (0.881, 0.970) | 0.001 |
| Respiratory rate (bpm) | 1.030 (1.026, 1.035) | ＜0.001 | 1.002 (0.995, 1.010) | 0.526 |

ICU: Intensive care unit; BMI: Body mass index; MICU: Medical intensive care unit; SOFA: Sequential organ failure assessment; CPD: Chronic pulmonary disease; CHF: Congestive heart failure; MAP: Mean arterial pressure

**Table S4.** The baseline characteristics of ICU patients between basophils-negative and basophils-positive groups after PSM.

| Variables | After PSM  (n=2114) | Basophils-negative  (n=1057) | Basophils-positive (n=1057) | *P* value |
| --- | --- | --- | --- | --- |
| Age (years) | 65 (54, 75) | 65 (54, 75) | 66 (54, 75) | 0.948 |
| Male, n (%) | 1254 (59.3) | 634 (60) | 620 (58.7) | 0.535 |
| BMI (kg/m^2^) | 27.99 (24.43, 33.07) | 27.92 (24.44, 32.97) | 28.08 (24.41, 33.27) | 0.896 |
| Admission type, n (%) |  |  |  | 0.562 |
| Elective | 598 (28.3) | 293 (27.7) | 305 (28.9) |  |
| Emergency/Urgent | 1516 (71.7) | 764 (72.3) | 752 (71.1) |  |
| SOFA score | 6 (4, 9) | 6 (4, 9) | 7 (4, 9) | 0.882 |
| Renal replacement therapy | 217 (10.3) | 109 (10.3) | 108 (10.2) | 0.943 |
| Noradrenaline use | 766 (36.2) | 376 (35.6) | 390 (36.9) | 0.526 |
| Mechanical ventilation | 1604 (75.9) | 805 (76.2) | 799 (75.6) | 0.760 |
| Service unit (MICU%) | 680 (32.2) | 346 (32.7) | 334 (31.6) | 0.576 |
| Laboratory tests |  |  |  |  |
| Bicarbonate | 24 (21.5, 26.30) | 23.89 (21.33, 26.34) | 24 (21.85, 26.21) | 0.334 |
| Hemoglobin | 9.64 (8.72, 10.84) | 9.63 (8.70, 10.83) | 9.64 (8.73, 10.85) | 0.981 |
| Lactate | 1.79 (1.3, 2.45) | 1.8 (1.3, 2.52) | 1.74 (1.3, 2.4) | 0.176 |
| PCO2 | 40.19 (36.5, 44.23) | 40.14 (36.26, 44.14) | 40.29 (36.75, 44.43) | 0.518 |
| Ph | 7.39 (7.35, 7.42) | 7.39 (7.35, 7.42) | 7.39 (7.36, 7.42) | 0.624 |
| Urea-nitrogen | 21 (14.33, 34.87) | 22 (15.2, 36) | 19.75 (13.5, 33.32) | 0.002 |
| White blood cell | 12.1 (9.3, 15.32) | 12.02 (9.05, 15.54) | 12.16 (9.54, 15.07) | 0.538 |
| Potassium | 4.1 (3.87, 4.4) | 4.10 (3.88, 4.4) | 4.11 (3.86, 4.41) | 0.805 |
| Sodium | 138.6 (136.3, 141.1) | 138.7 (136.4, 141.1) | 138.5 (136.2, 141.0) | 0.591 |
| Comorbidities, (n%) |  |  |  |  |
| Hypertension | 513 (24.3) | 252 (23.8) | 261 (24.7) | 0.648 |
| CPD | 565 (26.7) | 295 (27.9) | 270 (25.5) | 0.219 |
| Coronary disease | 784 (37.1) | 399 (37.7) | 385 (36.4) | 0.528 |
| CHF | 657 (31.1) | 342 (32.4) | 315 (29.8) | 0.205 |
| Cancer | 237 (11.2) | 114 (10.8) | 123 (11.6) | 0.535 |
| Liver disease | 309 (14.6) | 159 (15) | 150 (14.2) | 0.580 |
| Renal disease | 443 (21) | 227 (21.5) | 216 (20.4) | 0.557 |
| Cerebrovascular disease | 297 (14) | 151 (14.3) | 146 (13.8) | 0.754 |
| Vital signs |  |  |  |  |
| MAP (mmHg) | 58 (51, 81.5) | 58 (51, 82.5) | 58 (52, 80.5) | 0.870 |
| Heart rate (bpm) | 104 (90, 118) | 103 (90, 119) | 105 (91, 118) | 0.736 |
| Temperature (℃) | 36.39 (35.8, 37.56) | 36.39 (35.78, 37.59) | 36.39 (35.8, 37.46) | 0.922 |
| Respiratory rate (bpm) | 27 (24, 32) | 28 (24, 32) | 27 (24, 31.25) | 0.137 |

ICU: Intensive care unit; PSM: Propensity score-based matching; BMI: Body mass index; MICU: Medical intensive care unit; SOFA: Sequential organ failure assessment; CPD: Chronic pulmonary disease; CHF: Congestive heart failure; MAP: Mean arterial pressure

**Table S5.** Secondary outcome analyses with propensity score-matched cohorts.

| Secondary outcomes | Basophils-negative | Basophils-positive | *P* value |
| --- | --- | --- | --- |
| Norepinephrine use | 938 (48.60%) | 820 (42.4%) | ＜0.001 |
| Mechanical ventilation use | 1545 (80%) | 1460 (75.60%) | 0.001 |
| ICU duration | 6.51 (2.88, 12.56) | 4.36 (2.19, 8.85) | ＜0.001 |
| Hospital duration | 13.09 (7.74, 21.98) | 10.52 (6.19, 18.03) | ＜0.001 |

ICU: Intensive care unit

**Table S6.** The primary diagnosis of ICU admission.

| Primary diagnosis | Proportion |
| --- | --- |
| Sepsis | 13116 (59.45%) |
| Infectious | 31 (0.14%) |
| Cardiovascular | 3174 (14.39%) |
| Respiratory | 723 (3.28%) |
| Cancer | 404 (1.83%) |
| Gastrointestinal | 734 (3.33%) |
| Genitourinary | 362 (1.64%) |
| Trauma | 236 (1.07%) |
| Treatment complication | 203 (0.92%) |
| Neurological disease | 1182 (5.36%) |
| Dermopathy | 76 (0.34%) |
| Hemopathy | 225 (1.02%) |
| Endocrine metabolic diseases | 248 (1.12%) |
| Rheumatism | 24 (0.11%) |
| Other | 1324 (6.00%) |

**Table S7.** The baseline characteristics of septic patients between basophils-negative and basophils-positive groups.

| Variables | Basophils-negative | Basophils-positive | *P* value |
| --- | --- | --- | --- |
| Age (years) | 64 (52, 75) | 66 (55, 75) | ＜0.001 |
| Male, n (%) | 2750 (55.1) | 4776 (58.5) | ＜0.001 |
| BMI (kg/m^2^) | 28.0 (24.24, 33.10) | 28.52 (24.74, 33.39) | 0.027 |
| Admission type, n (%) |  |  | ＜0.001 |
| Elective | 781 (15.7) | 2089 (25.7) |  |
| Emergency/Urgent | 4209 (84.3) | 6037 (74.3) |  |
| SOFA score | 9 (6, 13) | 6 (4, 9) | ＜0.001 |
| Renal replacement therapy | 875 (17.5) | 571 (7) | ＜0.001 |
| Noradrenaline use | 2655 (53.2) | 2312 (28.5) | ＜0.001 |
| Mechanical ventilation | 3234 (64.8) | 4736 (58.3) | ＜0.001 |
| Service unit (MICU%) | 3084 (61.8) | 3207 (39.5) | ＜0.001 |
| Laboratory tests |  |  |  |
| Bicarbonate | 23 (20.11, 26) | 24.08 (22.06, 26.34) | ＜0.001 |
| Hemoglobin | 9.31 (8.44, 10.4) | 9.79 (8.82, 11) | ＜0.001 |
| Lactate | 1.93 (1.37, 2.81) | 1.7 (1.3, 2.29) | ＜0.001 |
| PCO2 | 40.04 (35.98, 44.67) | 40.57 (37.16, 44.63) | ＜0.001 |
| Ph | 7.38 (7.33, 7.41) | 7.39 (7.36, 7.42) | ＜0.001 |
| Urea-nitrogen | 30.51 (18.5, 48.79) | 19.67 (13.94, 31) | ＜0.001 |
| White blood cell | 13.38 (9.77, 18.25) | 12.03 (9.53, 14.9) | ＜0.001 |
| Potassium | 4.06 (3.83, 4.34) | 4.15 (3.89, 4.43) | ＜0.001 |
| Sodium | 139.24 (136.44, 142) | 138.68 (136.5, 141.33) | 0.647 |
| Comorbidities, (n%) |  |  |  |
| Hypertension | 1252 (25.1) | 2282 (28.1) | ＜0.001 |
| CPD | 1270 (25.5) | 2189 (26.9) | 0.061 |
| Coronary disease | 1027 (20.6) | 2904 (35.7) | ＜0.001 |
| CHF | 1367 (27.4) | 2543 (31.3) | ＜0.001 |
| Cancer | 1196 (24) | 910 (11.2) | ＜0.001 |
| Liver disease | 1069 (21.4) | 1138 (14) | ＜0.001 |
| Renal disease | 1078 (21.6) | 1782 (21.9) | 0.660 |
| Cerebrovascular disease | 560 (11.2) | 1172 (14.4) | ＜0.001 |
| Vital signs |  |  |  |
| MAP (mmHg) | 56 (48, 78) | 58 (52, 81.5) | ＜0.001 |
| Heart rate (bpm) | 114 (98.25, 130) | 101 (89, 116) | ＜0.001 |
| Temperature (℃) | 36.61 (35.78, 37.94) | 36.39 (35.8, 37.5) | ＜0.001 |
| Respiratory rate (bpm) | 30 (25, 34) | 27 (23, 31) | ＜0.001 |

BMI: Body mass index; MICU: Medical intensive care unit; SOFA: Sequential organ failure assessment; CPD: Chronic pulmonary disease; CHF: Congestive heart failure; MAP: Mean arterial pressure

**Table S8.** Types and percentage of main pathogenic bacteria in septic patients.

| Pathogenic bacteria | Basophils-negative | Basophils-positive | *P* value |
| --- | --- | --- | --- |
| Fungus, (n%) |  |  |  |
| Yeast | 4173 (11.87) | 2174 (7.27) | ＜0.001 |
| Mold | 200 (0.57) | 47 (0.16) | ＜0.001 |
| Gram Positive Bacteria, (n%) |  |  |  |
| Staph Aureus Coag+ | 6552 (18.64) | 7093 (23.71) | ＜0.001 |
| Streptococcus Pneumoniae | 251 (0.71) | 257 (0.86) | 0.036 |
| Enterococcus sp. | 1529 (4.35) | 1159 (3.87) | 0.002 |
| Viridans Streptococci | 69 (0.20) | 96 (0.32) | 0.002 |
| Clostridium Species | 192 (0.55) | 136 (0.45) | 0.101 |
| Gram Negative Bacteria, (n%) |  |  |  |
| Enterobacteriaceae | 7857 (22.35) | 8090 (27.05) | ＜0.001 |
| Pseudomonas Aeruginosa | 3720 (10.58) | 2272 (7.60) | ＜0.001 |
| Klebsiella Pneumoniae | 4101 (11.66) | 2995 (10.01) | ＜0.001 |
| Poteus Species | 528 (1.50) | 712 (2.38) | ＜0.001 |
| Bacteroides Fragilis Group | 98 (0.28) | 43 (0.14) | ＜0.001 |
| Acinetobacter sp. | 795 (2.26) | 468 (1.56) | ＜0.001 |
| Other |  |  |  |

**Table S9.** Univariate and multivariate models assessing the impact of basophils status and other clinical factors on 28-day mortality in septic patients.

| Variables | Univariate model | | Multivariate model | |
| --- | --- | --- | --- | --- |
|  | Odds Ratio  (95% CI) | *P* value | Odds Ratio  (95% CI) | *P* value |
| Basophils presence | 0.386 (0.352, 0.423) | ＜0.001 | 0.812 (0.693, 0.950) | 0.009 |
| Age | 1.015 (1.012, 1.018) | ＜0.001 | 1.024 (1.019, 1.030) | ＜0.001 |
| Gender/male | 0.912 (0.833, 0.998) | 0.045 | 0.822 (0.706, 0.957) | 0.012 |
| BMI | 0.989 (0.982, 0.997) | 0.005 | 0.980 (0.971, 0.990) | ＜0.001 |
| Admission type | 2.191 (1.923, 2.496) | ＜0.001 | 1.837 (1.508, 2.239) | ＜0.001 |
| SOFA score | 1.239 (1.225, 1.253) | ＜0.001 | 1.003 (0.979, 1.027) | 0.818 |
| Renal replacement therapy | 3.280 (2.915, 3.690) | ＜0.001 | 1.301 (1.062, 1.593) | 0.011 |
| Noradrenaline use | 4.767 (4.329, 5.249) | ＜0.001 | 2.287 (1.909, 2.742) | ＜0.001 |
| Mechanical ventilation | 2.395 (2.160, 2.654) | ＜0.001 | 1.879 (1.499, 2.356) | ＜0.001 |
| Service unit (MICU%) | 1.667 (1.522, 1.825) | ＜0.001 | 1.069 (0.910, 1.257) | 0.418 |
| Laboratory tests |  |  |  |  |
| Bicarbonate | 0.850 (0.841, 0.860) | ＜0.001 | 0.780 (0.749, 0.814) | ＜0.001 |
| Hemoglobin | 0.938 (0.913, 0.964) | ＜0.001 | 1.089 (1.038, 1.142) | ＜0.001 |
| Lactate | 1.758 (1.694, 1.824) | ＜0.001 | 1.474 (1.389, 1.565) | ＜0.001 |
| PCO2 | 1.004 (0.999, 1.009) | 0.141 | 1.133 (1.110, 1.156) | ＜0.001 |
| Ph | 0 | ＜0.001 | 554.545 (54.377, 5655.367) | ＜0.001 |
| Urea-nitrogen | 1.025 (1.023, 1.027) | ＜0.001 | 1.014 (1.010, 1.017) | ＜0.001 |
| White blood cell | 1.030 (1.025, 1.035) | ＜0.001 | 1.011 (1.003, 1.019) | 0.008 |
| Potassium | 2.100 (1.917, 2.300) | ＜0.001 | 1.036 (0.856, 1.253) | 0.720 |
| Sodium | 1.011 (1.001, 1.021) | 0.028 | 1.015 (0.998, 1.032) | 0.078 |
| Comorbidities |  |  |  |  |
| Hypertension | 0.768 (0.691, 0.853) | ＜0.001 | 0.947 (0.786, 1.141) | 0.567 |
| CPD | 1.170 (1.059, 1.292) | 0.002 | 1.074 (0.911, 1.266) | 0.397 |
| Coronary disease | 0.732 (0.661, 0.811) | ＜0.001 | 0.756 (0.634, 0.902) | 0.002 |
| CHF | 1.226 (1.114, 1.349) | ＜0.001 | 1.172 (0.993, 1.384) | 0.060 |
| Cancer | 2.031 (1.823, 2.263) | ＜0.001 | 1.856 (1.529, 2.252) | ＜0.001 |
| Liver disease | 2.242 (2.018, 2.492) | ＜0.001 | 1.631 (1.354, 1.965) | ＜0.001 |
| Renal disease | 1.309 (1.180, 1.453) | ＜0.001 | 0.905 (0.745, 1.099) | 0.313 |
| Cerebrovascular disease | 1.449 (1.282, 1.638) | ＜0.001 | 2.664 (2.194, 3.234) | ＜0.001 |
| Vital signs |  |  |  |  |
| MAP (mmHg) | 0.992 (0.990, 0.994) | ＜0.001 | 1 (0.998, 1.002) | 0.922 |
| Heart rate (bpm) | 1.011 (1.010, 1.013) | ＜0.001 | 1.005 (1.002, 1.008) | 0.001 |
| Temperature (℃) | 0.815 (0.788, 0.842) | ＜0.001 | 0.9 (0.856, 0.947) | ＜0.001 |
| Respiratory rate (bpm) | 1.024 (1.019, 1.029) | ＜0.001 | 1.001 (0.993, 1.009) | 0.805 |

BMI: Body mass index; MICU: Medical intensive care unit; SOFA: Sequential organ failure assessment; CPD: Chronic pulmonary disease; CHF: Congestive heart failure; MAP: Mean arterial pressure

**Table S10.** Primary outcome analyses with 5 different models elucidating the role of basophils absence in septic patients.

| Methods | Odds Ratio  (95% CI) | *P* value | Adjusted Odds Ratio (95% CI) | Adjusted *P* value |
| --- | --- | --- | --- | --- |
| Multivariate after multiple imputation | 1.170 (1.037, 1.318) | 0.010 | 1.832 (1.414, 2.370) | ＜0.001 |
| Doubly robust with all covariates | 1.220 (1.043, 1.427) | 0.013 | 1.629 (1.245, 2.128) | ＜0.001 |
| Doubly robust with unbalanced covariates | 1.218 (1.042, 1.422) | 0.013 | 1.623 (1.244, 2.398) | ＜0.001 |
| Propensity score matching | 1.166 (1.002, 1.353) | 0.046 | 1.634 (1.290, 2.075) | ＜0.001 |
| Propensity score IPW | 1.075 (1.001, 1.163) | 0.047 | 1.318 (1.200, 1.447) | ＜0.001 |

IPW: Inverse probability weighting

**Table S11.** Secondary outcome analyses with propensity score-matched cohorts.

| Secondary outcomes | Basophils-negative | Basophils-positive | *P* value |
| --- | --- | --- | --- |
| Norepinephrine use | 1107 (56.6%) | 982 (50.20%) | ＜0.001 |
| Mechanical ventilation use | 1637 (83.7%) | 1592 (81.4%) | ＜0.001 |
| Secondary acquired infections | 766 (28.97%) | 613 (21.71%) | ＜0.001 |
| ICU duration | 7.80 (3.91, 13.79) | 5.84 (3.05, 10.47) | ＜0.001 |
| Hospital duration | 14.20 (8.22, 23.84) | 11.83 (6.92, 20.12) | ＜0.001 |

ICU: Intensive care unit

**Table S12.** Univariate and multivariate models assessing the impact of basophils status and NLR on 28-day mortality in ICU patients with sepsis.

| Variables | Univariate model | | Multivariate model | |
| --- | --- | --- | --- | --- |
|  | Odds Ratio  (95% CI) | *P* value | Odds Ratio  (95% CI) | *P* value |
| Basophils absence | 2.591 (2.364, 2.841) | ＜0.001 | 1.232 (1.053, 1.443) | 0.009 |
| NLR | 1.018 (1.015, 1.022) | ＜0.001 | 1.010 (1.004, 1.016) | 0.001 |

NLR: Neutrophil-to-lymphocyte ratio; ICU: Intensive care unit

**Table S13.** Primary outcome analyses with 5 different models elucidating the role of NLR in ICU patients with sepsis.

| Methods | Adjusted Odds Ratio (95% CI) | Adjusted *P* value |
| --- | --- | --- |
| Multivariate after multiple imputation | 1.979 (1.409, 2.779) | ＜0.001 |
| Doubly robust with all covariates | 1.908 (1.364, 2.674) | ＜0.001 |
| Doubly robust with unbalanced covariates | 1.890 (1.355, 2.639) | ＜0.001 |
| Propensity score matching | 1.637 (1.197, 2.240) | ＜0.001 |
| Propensity score IPW | 1.742 (1.275, 2.379) | ＜0.001 |

NLR: Neutrophil-to-lymphocyte ratio; ICU: Intensive care unit; IPW: Inverse probability weighting
